# *Mycobacterium tuberculosis* ESAT-6 is a leukocidin causing Ca^2+^ influx, necrosis and neutrophil extracellular trap formation

**DOI:** 10.1038/cddis.2014.394

**Published:** 2014-10-16

**Authors:** R J Francis, R E Butler, G R Stewart

**Affiliations:** 1 Department of Microbial and Cellular Sciences and Bioimaging and Flow Cytometry Core Facility, Faculty of Health and Medical Sciences, University of Surrey, Guildford, GU2 7XH, UK

## Abstract

*Mycobacterium tuberculosis* infection generates pulmonary granulomas that consist of a caseous, necrotic core surrounded by an ordered arrangement of macrophages, neutrophils and T cells. This inflammatory pathology is essential for disease transmission and *M. tuberculosis* has evolved to stimulate inflammatory granuloma development while simultaneously avoiding destruction by the attracted phagocytes. The most abundant phagocyte in active necrotic granulomas is the neutrophil. Here we show that the ESAT-6 protein secreted by the ESX-1 type VII secretion system causes necrosis of the neutrophils. ESAT-6 induced an intracellular Ca^2+^ overload followed by necrosis of phosphatidylserine externalised neutrophils. This necrosis was dependent upon the Ca^2+^ activated protease calpain, as pharmacologic inhibition prevented this secondary necrosis. We also observed that the ESAT-6 induced increase in intracellular Ca^2+^, stimulated the production of neutrophil extracellular traps characterised by extruded DNA and myeloperoxidase. Thus we conclude that ESAT-6 has a leukocidin function, which may facilitate bacterial avoidance of the antimicrobial action of the neutrophil while contributing to the maintenance of inflammation and necrotic pathology necessary for granuloma formation and TB transmission.

Tuberculosis (TB) caused by *Mycobacterium tuberculosis* remains a leading source of mortality by infectious disease, with one-third of the world's population infected, 8.6 million new cases of TB and 1.3 million deaths annually.^1^ The fundamental feature of TB transmission is the generation of a pulmonary tubercle lesion that contains a cuff of immune cells surrounding a necrotic core laden with extracellular bacteria. This lesion may ‘cavitate' into the airways of the lung releasing the bacteria to allow transmission via the respiratory route. The essential contribution of macrophage and neutrophil cell death to the generation of this pathology has been recognised in many studies; however, notably in a comprehensive study of TB lesion development, Medlar^2^ observed that the polymorphonuclear cells were attracted to lesions following the death of mononuclear cells and that the bulk of necrotic tissue in human caseating tubercle lesions represented dead polymorphonuclear cells.

We now know that the bacterium has evolved mechanisms to regulate the mode and timing of macrophage cell death.^3, 4, 5, 6, 7^ After initial infection into the lungs, it is supposed that the bacterium is phagocytosed by alveolar macrophages which migrate into the interstitium of the lung.^8^ The bacterium is able to replicate intracellularly in the macrophage, inhibiting apoptosis until at a certain bacterial load it induces necrosis of the macrophage.^9^ The ensuing inflammation attracts monocytes and neutrophils from post-capillary venules that engulf the released bacteria, and thus sequential rounds of replication and inflammation enable the generation of the tubercle lesion. However, although we are beginning to understand the mechanisms of macrophage cell death control,^4, 5, 6^ we know very little about how *M. tuberculosis* modulates neutrophil death.

It is also clear that in some circumstances neutrophils have an antimycobacterial capacity,^10,11^ which may be mediated by the direct generation of reactive oxygen species (ROS) or by apoptosis of the infected neutrophils and subsequent efferocytosis of the apoptotic body combined with ROS-dependent killing.^12,13^ Additionally, neutrophil apoptosis has been linked to effective generation of adaptive immunity in *M. tuberculosis* infection.^14^ However, to counter this, *M. tuberculosis* has been recently shown to inhibit neutrophil apoptosis^14^ and furthermore, has been observed to induce necrosis.^11,12^ Interestingly, neutrophil necrosis only occurs on exposure to virulent strains which express the region of difference 1 (RD1) which encodes a type VII secretion system (ESX) that secretes proteins including the abundant early secretory antigen-6 (ESAT-6).^12,15, 16, 17^ Thus the bacterial induction of pro-inflammatory neutrophil necrosis may have dual benefit to the pathogen by removing the antimicrobial threat of the neutrophil while simultaneously facilitating the generation of the necrotic cavitating lesions that drive TB transmission.

The mechanism of necrosis in neutrophils can be varied and controlled. The most recent to be described is ‘NETosis',^18,19^ whereby death of the neutrophil results in formation of a structure made of DNA with a histone backbone which contains neutrophil elastase, myeloperoxidase (MPO) and metalloproteinases. These ‘traps' are known to be produced *in vivo* and associate with bacteria in some infections.^18,20, 21, 22^ Importantly they have been shown to be produced by *M. tuberculosis* infected neutrophils *in vitro*^23^ although with no bactericidal activity. As well as ‘NETosis' there are also other described mechanisms of neutrophil death, one of which is ‘secondary necrosis'. *In vitro* aging, without any stimuli, results in the necrosis of neutrophils that have undergone apoptosis (termed secondary necrosis). Previous investigations have shown that neutrophils that have externalised phosphatidylserine, and are therefore ‘apoptotic', can undergo secondary necrosis caused by a Ca^2+^ influx leading to the activation of a subtype of Ca^2+^ activated protease, calpain.^24^ This we termed Ca^2+^ Induced Necrosis (CAIN). In the present study, we elucidate the molecular events that link the RD1 encoded ESX-1 type VII secretion system with neutrophil necrosis. We chose to investigate the ESAT-6 protein, which is secreted by ESX-1, because it interacts with lipid membranes and is thought to be pore-forming,^25, 26, 27, 28^ thus it has the potential to influence intracellular Ca^2+^. Furthermore, calpain has been shown previously to be active in *M.bovis* infections that were dependent on the RD1 locus.^29^ ESAT-6 has also been shown to have cytotoxic effects to pneumocytes^30^ and T lymphocytes.^31^ Therefore, this study aimed to determine if ESAT-6 had a leukocidin action, and if so, whether this was dependent on intracellular flux of Ca^2+^, activation of calpain, and further, if this resulted in the formation of neutrophil extracellular traps (NETs).

## Results

### ESAT-6 protein increases intracellular Ca^2+^ and subsequent necrosis in phosphatidylserine externalised neutrophils

**Figure 1:**
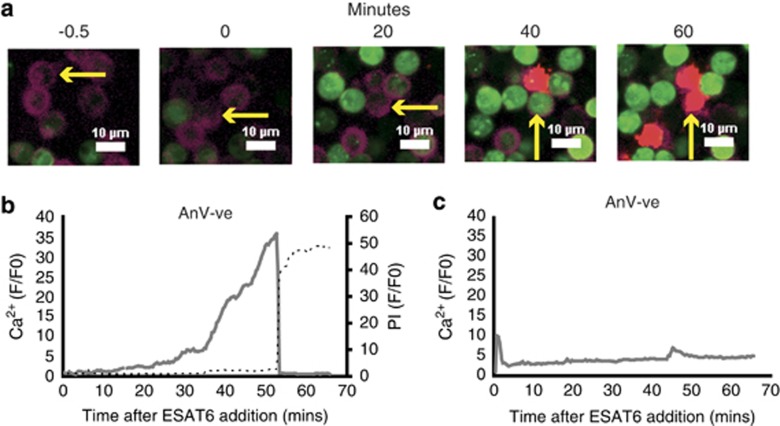
Intracellular Ca^2+^ increases in ESAT-6 treated neutrophils and precedes necrosis. (**a**) Sequential fluorescence micrographs showing accumulation of calcium (green, Fluo4) in Annexin V positive (purple)/PS externalised aged neutrophils after exposure to exogenous ESAT-6 (20 *μ*g/ml). Uptake of propidium iodide (PI) (red) indicates necrosis of the cell. Arrow tracks an individual cell. White bar=10 *μ*m. Panel (**b**) shows a representative quantitation of intracellular Ca^2+^ levels of a PS externalised (Annexin V+ve) cell exposed to ESAT-6 (16 responses in *N*=3 experiments). Panel (**c**) shows representative intracellular Ca^2+^ in a non-PS externalised neutrophil exposed to ESAT-6. (21 responses in *N*=4)

Previous work had identified increased intracellular Ca^2+^ as a mechanism of secondary necrosis in human neutrophils.^24^ To examine the effect of ESAT-6 on neutrophil Ca^2+^ we loaded 24- h aged cells with fluo4-AM, a fluorescent Ca^2+^ indicator that stays within intact cells following uptake and conversion from its AM ester to the hydrophilic fluo4 molecule.^32^ Using confocal microscopy we observed that the addition of ESAT-6 caused an increase in intracellular Ca^2+^ levels in a proportion of neutrophils within 20 min of exposure. The sequential micrographs in Figure 1a illustrate the Ca^2+^ influx as increasing Fluo4 fluorescence (green) in a phosphatidylserine externalised (Annexin V positive, purple) cell that ultimately undergoes necrosis (propidium iodide, red) at 60 min post-ESAT-6 addition. A movie of this response is available (Supplementary Figure 1). A representative quantitation of the Ca^2+^ increase followed by simultaneous Ca^2+^ release and uptake of propidium iodide at the point of cell lysis is shown in Figure 1b. Interestingly ESAT-6 did not induce significant calcium uptake in cells without externalised phosphatidylserine (annexin V negative) (Figure 1c).

**Figure 2:**
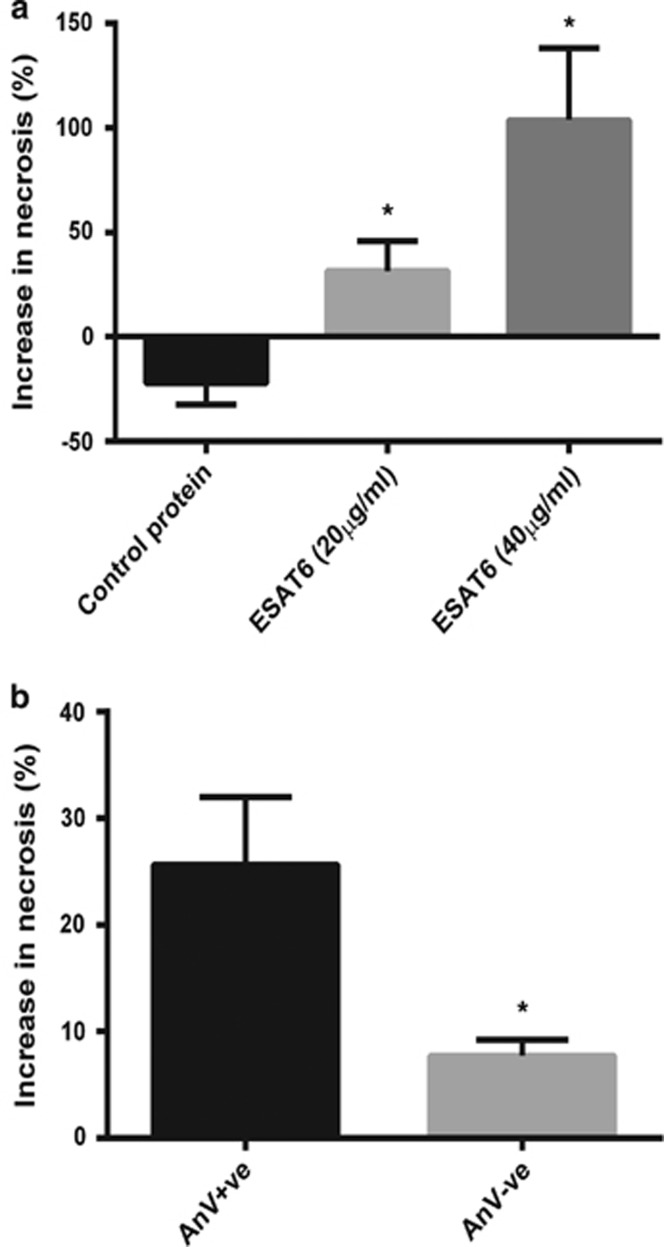
ESAT-6 causes selective necrosis of phosphatidylserine externalised neutrophils. (**a**) Addition of ESAT-6 causes an increase in the proportion of propidium iodide positive cells as assessed by flow-cytometry. Control protein is recombinant Rv0435c. Ionomycin is a known inducer of neutrophil necrosis. Data normalised to vehicle in each experiment. Accumulated data *N*>4 for each condition (three repeats per experiment). *P<0.05 using Student's unpaired *t* test compared to control protein. (**b**) The increase in necrosis caused by ESAT-6 (20 *μ*g/ml) occurred preferentially in PS externalised cells (Annexin V-FITC, AnV+ve). *N*=4 (in triplicate). *P<0.05 using unpaired Student's *t* test. Error bars are S.E.M.

To quantify the level of necrosis, we used flow cytometry to measure the percentage of propidium iodide positive cells 60 min after addition of ESAT-6 (Figure 2a). The increase in necrosis was dose dependent with 20 *μ*g/ml ESAT-6 causing a 25% increase in necrosis and 40 *μ*g/ml inducing 100% increase in neutrophil necrosis. As a positive control we treated neutrophils with the *Streptomyces conglobatus* ionophore, ionomycin, which is known to induce necrosis in neutrophils. Ionomycin induced a 150% increase in necrosis. Stimulation of aged neutrophils with a control recombinant *M. tuberculosis* protein (Rv0435c, chosen as a non-RD1/ESX-1 protein to control for non-specific effects of using a heterologously expressed protein) did not induce neutrophil necrosis. Further analysis by flow cytometry confirmed that necrosis was occurring preferentially in the annexin V positive neutrophils (Figure 2b). Thus we conclude that ESAT-6 causes necrosis of phosphatidylserine externalised neutrophils in a Ca^2+^ dependent manner.

### Calpain activation by Ca^2+^ influx causes necrosis in ESAT-6 stimulated phosphatidylserine externalised neutrophils

**Figure 3:**
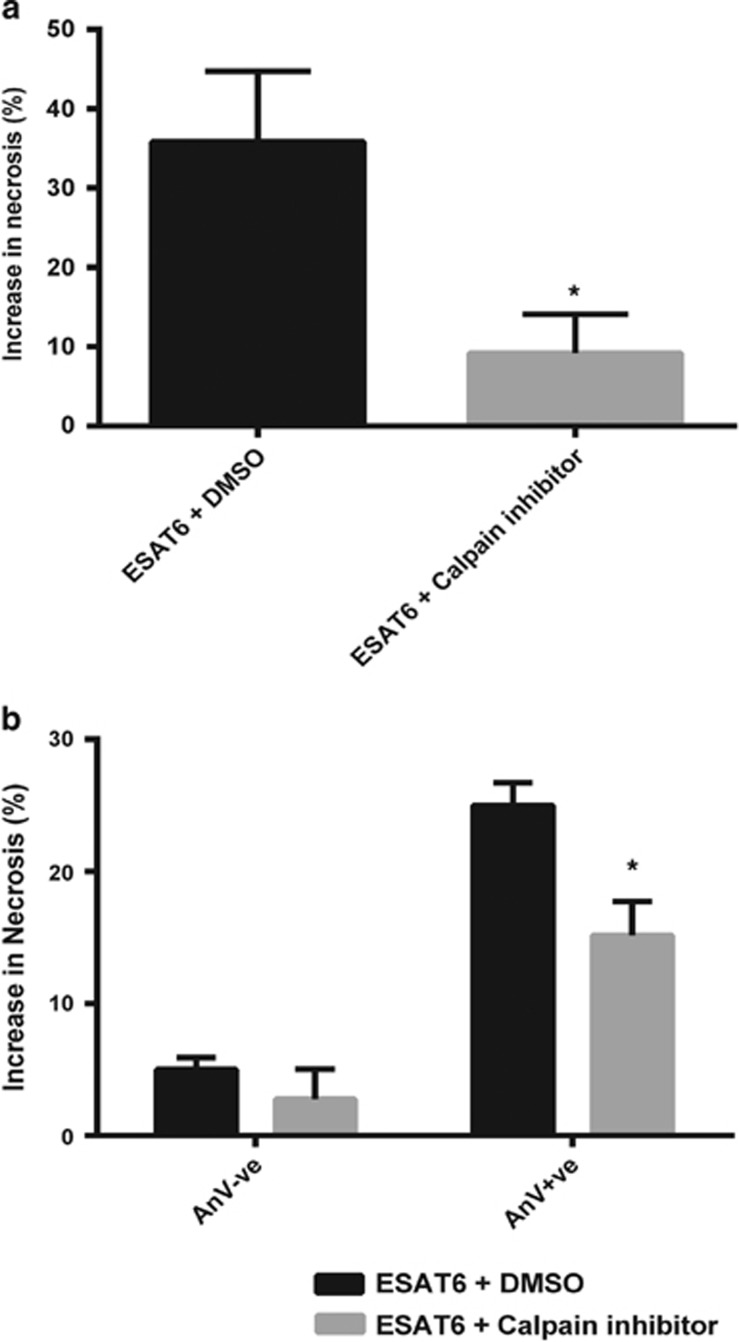
Calpain mediates ESAT-6 induced neutrophil necrosis. (**a**) Pre-treatment of neutrophils with the calpain inhibitor, PD150606, inhibits the induction of necrosis by ESAT-6 (20 *μ*g/ml). Necrosis was measured by flow-cytometry of propidium iodide uptake 1 h post-ESAT-6 stimulation. Data are normalised to vehicle plus DMSO. *N*=4 (in triplicate). *P<0.05. (**b**) The decrease in ESAT-6 dependent necrosis caused by calpain inhibition occurred preferentially in PS externalised cells (Annexin V-FITC, AnV +ve). Representative data *N*=3 (in triplicate). *P<0.05 using Student's *t* test

The Ca^2+^ activated cysteine proteases known as calpains have been previously implicated in the necrosis of neutrophils.^24^ To test if calpains were involved in necrosis of ESAT-6 stimulated neutrophils, we pre-incubated aged neutrophils with the pan-specific calpain inhibitor, PD150606,^33^ before stimulation with ESAT-6. The inhibitor significantly reduced the level of cell death induced by ESAT-6 (Figure 3a). Furthermore, the inhibitor reduced necrosis in those cells with externalised phosphatidylserine (Figure 3b). These results indicate that ESAT-6 can induce necrosis in the phosphatidylserine externalised population through the Ca^2+^ activated calpain family of proteases.

### Necrosis by ESAT-6 leads to the formation of NET-like structures

**Figure 4:**
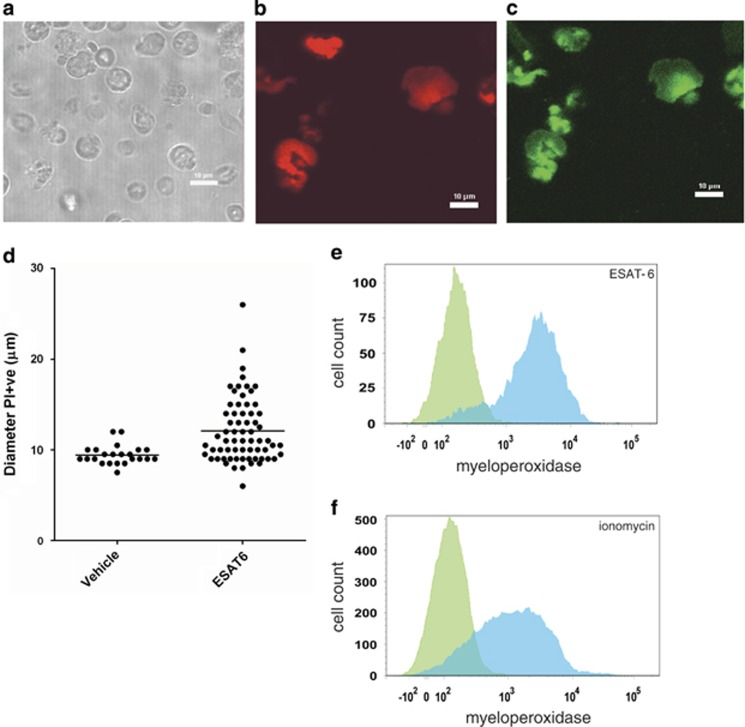
ESAT-6 dependent lysis releases NETs. (**a**) Neutrophils were treated with 40 *μ*g/ml ESAT-6 and the morphology and propidium iodide (PI) fluorescence (**b**) assessed by confocal microscopy (white bar=10 *μ*m). Propidium iodide positive cells have an expanded morphology consistent with lysis and extrusion of NET-like structures containing DNA. (**c**) MPO immunoreactivity of ESAT-6 lysed neutrophils confirms identity as NETs. (**d**) The mean diameter of necrotic ESAT-6 stimulated neutrophils is significantly greater than necrotic cells from cultures treated with vehicle alone (*P*<0.001 by T test, cells counted from greater than 10 fields of view). Flow cytometric quantitation of MPO externalisation in ESAT-6 treated neutrophils (**e**) shows similar high levels of MPO release into the NET as that observed with ionomycin (5 *μ*M) generated NETs (**f**); anti-MPO FITC (blue) compared with isotype control (green). Representative data from two donors

NET formation has often been seen during necrosis of neutrophils, so we tested whether NETs were produced during neutrophil necrosis induced by ESAT-6. We used confocal scanning laser microscopy to analyse the morphology and composition of the ESAT-6 treated and observed that they had an expanded structure (Figure 4) and stained positively with propidium iodide, indicative of extruded DNA consistent with previous observations of NETs (Figures 4a and b). The ESAT-6 treated cells had a larger diameter compared to cells treated with vehicle alone (Figure 4d). NETs are also known to contain MPO,^34^ so to further confirm the identity of the ESAT-6 induced necrotic population we assessed the presence of exposed MPO using confocal microscopy (Figure 4c) and flow cytometry (Figure 4e) with FITC labelled antibodies to MPO. This showed that MPO was widely accessible and associated with the ESAT-6-induced NET-like cell population. This localisation of MPO to the ESAT-6 NETs was similar to that observed when neutrophils were treated with ionomycin (Figure 4f). Ionomycin stimulates calcium influx across the cell membrane and thus ESAT-6 and ionomycin may stimulate NETosis through similar pathways.

## Discussion

The ESX-1 secretion system is an essential requirement for *M. tuberculosis* virulence. Its deletion from the genome of *M. bovis* BCG represents a key step in the attenuation of the vaccine strain and despite being perhaps the most studied molecular system in mycobacteria, its functions remain elusive but may be many.^35^ It secretes a number of proteins, the most prominent of which are ESAT-6 and its putative partner Cfp10, with which it forms a heterodimer.^36^ If there is one predominant theme in the proposed functions of ESAT-6 it is that it interacts with lipid membranes.^25, 26, 27, 28^ One result of this may be disruption of the phagosome membrane allowing *M. tuberculosis* to escape from its phagosome into the cytoplasm of mononuclear phagocytic cells.^37^ This phagosome rupture has itself been linked to the generation of cell death, possibly by the activation of NLRP3.^38, 39, 40^ ESAT-6 is however secreted in great quantity even in the extracellular environment. In fact, Cfp10 and ESAT-6 represent two of the most abundant proteins found in short-term culture supernatants of axenic broth culture.^41,42^ Thus, whether inside or outside of a host cell, secreted ESAT-6 has the opportunity to interact with both the internal and external membranes of the cell. This may be of particular relevance to development of the necrotic granuloma during which there is considerable neutrophil and monocyte death and *M. tuberculosis* is located both intra- and extracellularly.^43^

In support of ESAT-6 having an important extracellular function, we show in the present study and for the first time that exogenous ESAT-6 has a leukocidin-like action, inducing Ca^2+^ influx in aging neutrophils leading to calpain activation and necrosis. Thus ESAT-6 induced necrosis appears to occur by a similar mechanism to ionophore induced necrosis of neutrophils.^24^ Exactly how ESAT-6 induces Ca^2+^ movement across the cell membrane is unknown but previously others have suggested that it forms membrane pores.^26^ Indeed, pore formation is a common mechanism used by pathogenic bacteria to lyse host cells—for instance *Escherichia coli* haemolysins,^44^ *Listeria* listeriolysins^45^ and *Streptococcus pyogenes* streptolysins O and S.^46^ The ESAT-6/Cfp10 complex does not have the electrostatic surface properties typical of pore forming proteins and appears to bind to cell surface receptors.^36^ However, there is good evidence that ESAT-6 can dissociate from Cfp10 in certain conditions such as acidity,^12^ can undergo conformational change in the presence of phospholipids^47^ and can directly interact with membrane structure.^25,28^

It was striking that ESAT-6 specifically raised cytosolic Ca^2+^ levels and necrosis in phosphatidylserine externalised neutrophils with a negligible effect on cells without phosphatidylserine externalised. We hypothesise a number of explanations for this. Firstly, it is feasible that phosphatidylserine (PS) is a binding/interaction partner for ESAT-6 and thus membrane perturbation occurs only in PS externalised cells. Alternatively, the membrane repair mechanisms that confer resistance to bacterial and host derived membrane pores, such as endocytosis and lysosomal degradation of pore forming proteins^48^ may be less effective in PS externalised neutrophils. This is because effector proteins of these repair mechanisms including annexin A1 and synaptotagmin VII require PS to be available in the cytosolic leaflet of the cell membrane to catalyse their localisation to the cell membrane via their C2 domains. A similar mechanism may also explain the calpain dependence of necrosis that we observed, because calpain normally translocates to the cell surface upon activation in a process involving PS and elevated Ca^2+^.^49^ However, in cells with externalised PS there may be insufficient PS available in the cytosolic leaflet, thus the protease remains free to roam the cytosol in an uncontrolled but active proteolytic state resulting in necrosis.

We also used flow cytometry and confocal laser scanning microscopy to observe NET formation, demonstrating ESAT-6 treated necrotic neutrophils produced NETs. Induction of NETosis by pathogen derived membrane pores is a mechanism that has been identified recently, for example purified Panton–Valentine leukocidin (PVL) and leukotoxin GH from *Staphylococcus aureus*^50^ and leukotoxin from *Mannheimia haemolytica* caused NET formation.^51^ The mechanism of NETosis by bacterial toxins was previously unknown, but we provide evidence that, at least in the case of ESAT-6, it is Ca^2+^ dependent. This is supported by work from others that have showed calcium ionophore A23187^52^ and thapsigargin,^53^ which increases intracellular Ca^2+^ by inhibition of sarcoplasmic/endoplasmic ATPase (SERCA), can induce NET formation in a mechanism suggested to be through peptidylarginine deiminase (PAD) 4 hypercitrullination of histones.^52,54^ Indeed, we show that ESAT-6 and ionomycin treated neutrophils exhibited similar external localisation of MPO indicative of NET formation. NETosis has not been previously described as requiring PS externalisation.^19^ However, we have identified that, similar to secondary necrosis, ESAT-6 specifically induced NETosis in PS externalised cells.

Our observations that ESAT-6 causes Ca^2+^ dependent secondary necrosis or CAIN and NETosis provide a mechanistic basis for previous observations that virulent strains of *M. tuberculosis* carrying the ESX-1 secretion system can kill neutrophils when in co-culture.^12^ This leukocidin virulence mechanism may remove the antimycobacterial threat of the neutrophil while simultaneously generating nutrient rich necrotic tissue that fuels extracellular growth of the mycobacterium. This is consistent with observations of human tubercle lesions packed with dying neutrophils^2^ and more recent observations that the development of caseous, necrotic granulomas in a mouse model of progressive TB were associated with the presence of extracellular bacteria, neutrophil necrosis and NET-like structures.^55^ Thus ESAT-6 induction of NETs may be an important mechanism for the generation of lung pathology associated with active transmissible tuberculosis. There remains the possibility that other *M. tuberculosis* molecules can also induce neutrophil death because RD1 deleted mutants retain a residual cytotoxicity.^12^ Future work will aim to identify further mycobacterial effectors of neutrophil necrosis and establish if pharmacologic intervention of neutrophil necrosis alters pathology during infection.

## Materials and Methods

### Samples

Neutrophils were isolated from whole blood samples from healthy volunteers (BCG vaccinated) obtained through favourable ethical opinion by the University of Surrey Ethical Board.

### Reagents

Dextran and ficoll (Sigma-Aldrich, Gillingham, Dorset, UK) were used for neutrophil purification from whole blood. Recombinant ESAT-6 expressed in *Lactococcus lactis* (Statum Serum Institute, Denmark) was used for experiments. Recombinant *M. tuberculosis* protein Rv0435c was used as a non-ESX-1 related protein control. Ionomycin was added to cells at 5 *μ*M. Viability of neutrophils was measured using propidium iodide at 500 nM (Sigma-Aldrich). For flow cytometry, phosphatidylserine externalisation was detected using annexin V-FITC (2.5 *μ*g/ml) and for confocal laser scanning microscopy annexin V-alexa fluor 647 (2.5 *μ*g/ml) was used (BioLegend, San Diego, CA, USA). Fluo4-AM (10 *μ*M) was used to measure Ca^2+^ concentration (Invitrogen Life Technologies, Paisley, UK). FITC conjugated antibodies to MPO and isotype control (347201—BioLegend), were used for flow cytometric analysis of neutrophil death.

### Neutrophil preparation

Buffy coats were prepared by dextran sedimentation and haemolysis as previously described.^53,54^ For further purification, the resulting white blood cells were separated using a ficoll density gradient.^56^ The neutrophils were suspended in RPMI with 10% FCS, stable l-glutamine (0.29 mg/ml), penicillin (0.1 mg/ml), streptomycin (0.1 mg/ml), and FCS (10%) and maintained at 37 °C, 5% CO_2_ overnight, in cell culture flasks before treatments and further analysis. Before manipulation, neutrophil numbers were counted using a haemocytometer and purity determined (>95%) (NanoEnTek, Seoul, Korea).

### Measurement of neutrophil necrosis

At the beginning of each experiment, the viability and phosphatidylserine externalisation of the aged neutrophils was measured using propidium iodide and annexin V-FITC, respectively, by flow cytometry using a FACS Canto (Becton Dickinson, Oxford, UK). ESAT-6 (20 or 40 *μ*g/ml as stated), PBS vehicle or control protein was then added at the concentration stated in text. Proteins were added from a 2x stock solution to reduce locality artefacts. The increase in necrosis was then measured in each population as an increase in the percentage of propidium iodide positive neutrophils (as determined by gating). Where stated, the data was normalised to the increase in necrosis in vehicle only controls to enable comparisons between donors and expressed as a percentage increase.

### Confocal laser scanning microscopy of neutrophils

Images were produced on either a LSM 510 (Zeiss, Oberkochen, Germany) or A1M (Nikon, Tokyo, Japan) CLSM with the neutrophils kept in glass-bottomed petri dishes (MatTek, Ashland, MA, USA). Measurements were obtained of the fluo4-AM dyed neutrophils by same sized region of interest tracking through the time-course of the experiment with the mean fluorescence intensity (MFI) measured. This was divided by the MFI of time 0 (F/F_0_), or the closest recording to that point. To determine whether the neutrophils had externalised phosphatidylserine, they were also stained prior to stimulation with annexinV-fluor647. To confirm necrosis, we stained for extracellular DNA with propidium iodide. Images were analysed uniformly between controls and treatment using NIS Elements (Nikon) or Zen 2009 (Zeiss), and images produced using ImageJ (NIH).

### Localisation of myeloperoxidase in Neutrophil Extracellular Traps

We examined the extruded MPO content of NETs stimulated by the addition of ESAT-6 at 40 *μ*g/ml for 1 h or ionomycin at 5 *μ*M for 30 min. The cells were centrifuged at 1000rcf for 1 min to create a pellet. The media was removed and the neutrophils were resuspended in ‘blocking buffer' (5% heat inactivated normal human serum and 1% bovine serum albumin in PBS). The FITC-conjugated antibodies for MPO or the isotype control were added in PBS with block at 1 : 50 for 30 min. The neutrophils were centrifuged and resuspended in PBS ready for flow cytometric analysis or CLSM imaging.

### Statistical analysis

The results are presented as the mean+S.E.M. All statistics are Student's *t* test or ANOVA with Dunnett's post test as stated.

## Abbreviations

ANOVA: analysis of variance
AnV: annexin V
BCG: Bacille Calmette Guérin
Ca^2+^: calcium
CAIN: Ca^2+^ induced necrosis
Cfp10: culture filtrate protein 10 kDa
CLSM: confocal laser scanning microscopy
DMSO: dimethyl sulfoxide
ESAT-6: 6 kDa early secretory antigenic target
ESX-1: ESAT-6 secretion system-1
FCS: foetal calf serum
FITC: fluorescein isothiocyanate
MFI: mean fluorescence intensity
MPO: myeloperoxidase
NET: neutrophil extracellular trap
NLRP3: NOD-like receptor family, pyrin domain containing 3
PI: propidium iodide
PS: phosphatidylserine
RD1: Region of Difference 1
ROS: reactive oxygen species
S.E.M.: standard error of the mean
TB: tuberculosis
